# *De Novo* Transcriptomic Resources in the Brain of *Vespa velutina* for Invasion Control

**DOI:** 10.3390/insects11020101

**Published:** 2020-02-03

**Authors:** Miao Wang, Hanyu Li, Huoqing Zheng, Liuwei Zhao, Xiaofeng Xue, Liming Wu

**Affiliations:** 1 Institute of Apicultural Research, Chinese Academy of Agricultural Sciences, Beijing 100093, China; wangmiao03@caas.cn (M.W.); zhaoliuwei@caas.cn (L.Z.); xuexiaofeng@caas.cn (X.X.); 2 College of Animal Sciences and Technology, Huazhong Agricultural University, Wuhan 430070, China; lhy98@webmail.hzau.edu.cn; 3 College of Animal Science, Zhejiang University, Hangzhou 310058, China; hqzheng@zju.edu.cn

**Keywords:** *Vespa velutina*, hornet, invasion control, *de novo* transcriptome

## Abstract

The invasion of *Vespa velutina* presents a great threat to the agriculture economy, the ecological environment, and human health. An effective strategy for this hornet control is urgently required, but the limited genome information of *Vespa velutina* restricts the application of molecular-genomic tools for targeted hornet management. Therefore, we conducted large-scale transcriptome profiling of the hornet brain to obtain functional target genes and molecular markers. Using an Illumina HiSeq platform, more than 41 million clean reads were obtained and *de novo* assembled into 182,087 meaningful unigenes. A total of 56,400 unigenes were annotated against publicly available protein sequence databases and a set of reliable Simple Sequence Repeats (SSRs) and Single Nucleotide Polymorphisms (SNP) markers were developed. The homologous genes encoding crucial behavior regulation factors, odorant binding proteins (OBPs), and vitellogenin, were also identified from highly expressed transcripts. This study provides abundant molecular targets and markers for invasive hornet control and further promotes the genetic and molecular study of *Vespa velutina*.

## 1. Introduction

The yellow-legged hornet, *Vespa velutina*, is a damaging invader that causes negative ecological, economic, and social impacts [1]. This hornet originates from Asia and has made rapid and high-profile invasions since its introduction into Europe [2]. As an efficient predator of beneficial insects, such as the western honeybee, the hornet induces considerable damage to agribusiness by impacting pollination and apicultural [1]. Moreover, hornets represent a potential threat to biodiversity due to their overlapping ecological niches [3,4]. In addition, the severe immediate allergic reactions caused by hornet sting has long been recognized as a life-threatening risk for human health [5,6].

At present, the management of the threat posed by hornets largely relies on nest destruction or traps. Many kinds of techniques are currently being used to locate hornet nests, including harmonic radar [7,8,9] and radio-telemetry [10]. Several groups have been working on the identification of sex pheromones [11,12,13] and chemicals [14] to develop specific traps. Hornet behavior [4,15,16,17] and olfactory systems [13,18,19] are areas of active research for developing control strategies, but little is known about the molecular level characteristics of *Vespa velutina*. Although molecular markers, such as mitochondrial DNA sequences [20,21,22] and microsatellites [23], have already been used to identify *Vespa velutina* samples [24] and define their distribution area [25,26], molecular-genomic tools are rarely used for hornet control. The key design points for molecular-genomic approaches are the selection of target genes encoding essential proteins/enzymes of important physiological pathways [27]; however, the lack of genome information, functional gene annotation, and molecular markers restricts the employment of target genes against *Vespa velutina* to ensure a prolonged and sufficient incapacitation of the pest.

In the current investigation, we conducted a *de novo* transcriptome determination from the brain of *Vespa velutina*. In this nerve center that is responsible for hornet behavior, 182,087 unigenes were discovered and 56,400 unigenes were annotated to seven databases and assigned to a multitude of important physiological pathways. We also identified genes encoding proteins homologous to odorant binding proteins (OBPs) and vitellogenin (Vg), which play vital roles in various behaviors [28,29,30]. These data provide abundant targets of functional genes, and more than 100 million Simple Sequence Repeats (SSRs) and Single Nucleotide Polymorphisms (SNPs) were explored as marker resources for investigation of *Vespa velutina*. Our study improved the genomic knowledge of *Vespa velutina* at the transcriptomic level, and it provides genetic information and molecular targets for invasion control of *Vespa velutina*. 

## 2. Materials and Methods

### 2.1. Sampling and RNA Extraction

*Vespa velutina* specimens were captured near honeybee hives when hornets were hunting. All the hornet samples were collected at the same site in Hangzhou, China. The hornets were anaesthetized in ice-cold water, and the brains were subsequently dissected, followed by immersion in TRIzol Reagent (Invitrogen, Carlsbad, CA, USA). Six sample were collected with a single brain per sample. Total RNA from each sample was extracted using TRIzol reagent (Invitrogen). The RNA quality was analysed using a NanoPhotometer spectrophotometer (IMPLEN, Westlake Village, CA, USA) to assess the RNA purity, and the RNA Nano 6000 Assay Kit for the Agilent Bioanalyzer 2100 System (Agilent Technologies, Santa Clara, CA, USA) was used to assess RNA integrity. 

### 2.2. cDNA Library Construction and Illumina Sequencing

The sequencing libraries were generated using the NEB Next Ultra RNA Library Prep Kit for Illumina (NEB, Ipswich, MA, USA) according to the manufacturer’s instructions. Briefly, the mRNA was purified from total RNA using poly-T oligo-attached magnetic beads. Subsequently, short fragments of the enriched mRNA were generated and reverse transcribed into cDNA using the PrimeScript 1st Strand cDNA Synthesis Kit (TaKaRa, Beijing, China). These cDNA fragments were subjected to end repair and ligation with Illumina adapters. A total of six libraries with an insert size of 200 bp were constructed, and the library quality was assessed using the Agilent Bioanalyzer 2100 System. After cluster generation, the library preparations were sequenced on an Illumina HiSeq platform, and paired-end reads were generated.

### 2.3. De novo Assembly and Annotation

Transcriptome *de novo* assembly was performed using Trinity Software [31] with the min_kmer_cov set to 2, *k-mer* value of 25 and all default parameters. Quality of the assemblies was assessed using Benchmarking Universal Single-Copy Orthologs (BUSCO) software v3.0.2 [32,33] against the metazoa_odb9 dataset [33]. To run BUSCO, the programs ncbi-blast-2.7.1 and hmmer-3.1b2. were used. The sequences were annotated using a series of sequential BLAST searches designed to identify the most descriptive reads. The database, software, and parameters used in annotation are listed in Supplementary Table S1. 

### 2.4. Detection of SSR, SNP Markers

Picard-tools v1.41 and samtools v0.1.18 were used to sort, remove duplicated reads, and merge the bam alignment results of each sample. GATK2 software was used to perform SNP calling. Raw vcf files were filtered with the GATK standard filter method and other parameters (clusterWindowSize: 10; MQ0 ≥ 4 and (MQ0/(1.0*DP)) > 0.1; QUAL < 10; QUAL < 30.0 or QD < 5.0 or HRun > 5), and only SNPs with distance >5 were retained. SSR of the transcriptome were identified using MISA (microsatellite identification tool) [34], and primers for each SSR were designed using Primer3 [35]. 

### 2.5. Phylogenetic Analysis and Quantification of Gene Expression Levels

The amino acid sequences of VvOBP1-4, VvOr1-2, VvVg1-4, and VvVgr were used as queries to search for their homologous proteins using protein BLAST. The amino acid sequences were aligned with multiple sequence alignment Clustal Omega Web Services [36] with default parameter. Bayesian phylogenetic trees were constructed by MrBayes v3.2.7a [37] with mutation rate distribution model “Invgamma” and 1,000,000 generations analysis. The final phylogenetic tree was display by FigTree v1.4.4 [38] according to tree display recommendation within manual of MrBayes. Gene expression levels were estimated by RSEM (RNA-seq by expectation maximization) for each sample. Clean data were mapped back onto the assembled transcriptome and read count for each gene was obtained from the mapping results.

## 3. Results

### 3.1. Sequencing and De Novo Assembly of Vespa Velutina Transcriptomes 

**Figure 1:**
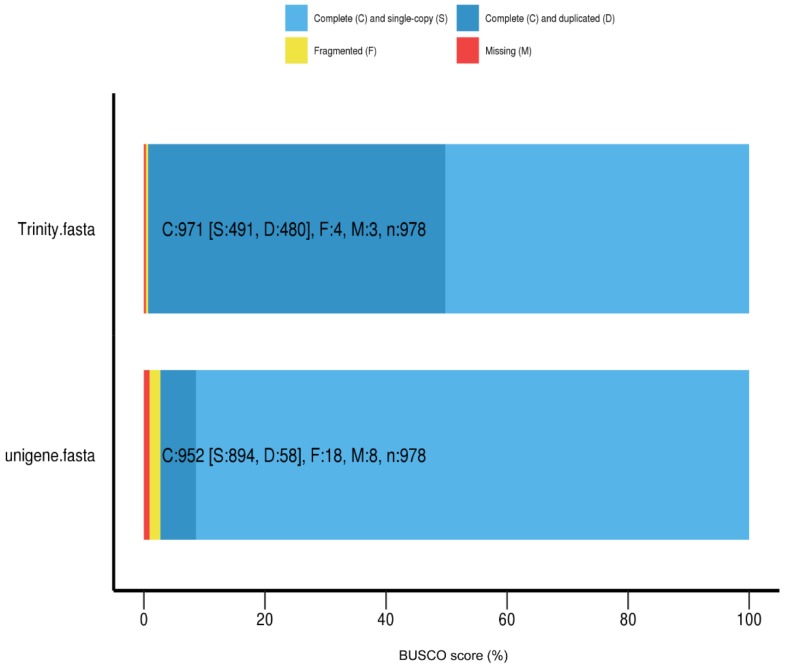
Transcriptome completeness of *de novo* assembled *Vespa velutina* transcripts and unigenes using Benchmarking Universal Single-Copy Orthologs (BUSCO).

Six *Vespa velutina* samples were used for Illumina sequencing with a single brain per sample. More than 47 million raw reads were generated for each of the six samples, totaling approximately 7 Gb of data. After adapter trimming and quality filtering, we obtained more than 41 million clean reads, with more than 91% of all bases having Phred (Q) scores higher than 30, and GC percent ranged from 37.34% to 38.03%. (Table S2). The clean reads were then *de novo* assembled by Trinity method using k-mer values of 25 (default). As shown in the Trinity dataset (Table S3: Additional file 1), 208,274 transcripts were obtained as reference sequence (Table S5). The longest of transcripts belonged to the same gene (generally the appropriate representative of the gene) is chosen as unigene (Table S4: Additional file 2), and 182,087 unigenes were consequently generated (Table S5). These transcripts and unigenes ranged in size from 201 to 26,255 bp, with an average size of 675 bp and 486 bp, respectively (Table S5). The N50 was recorded as 1315 and 522 for transcripts and unigenes, respectively (Table S5). Additionally, to evaluate the completeness of assemblies, the Benchmarking Universal Single-Copy Orthologs (BUSCO) analysis was performed on the Trinity and unigene datasets. The BUSCO complete scores were 99.3% and 97.3% for Trinity and Unigene, respectively, indicating a good quality of assemblies that can be used for further analysis (Figure 1 and Table S6).

### 3.2. Functional Annotation 

**Figure 2:**
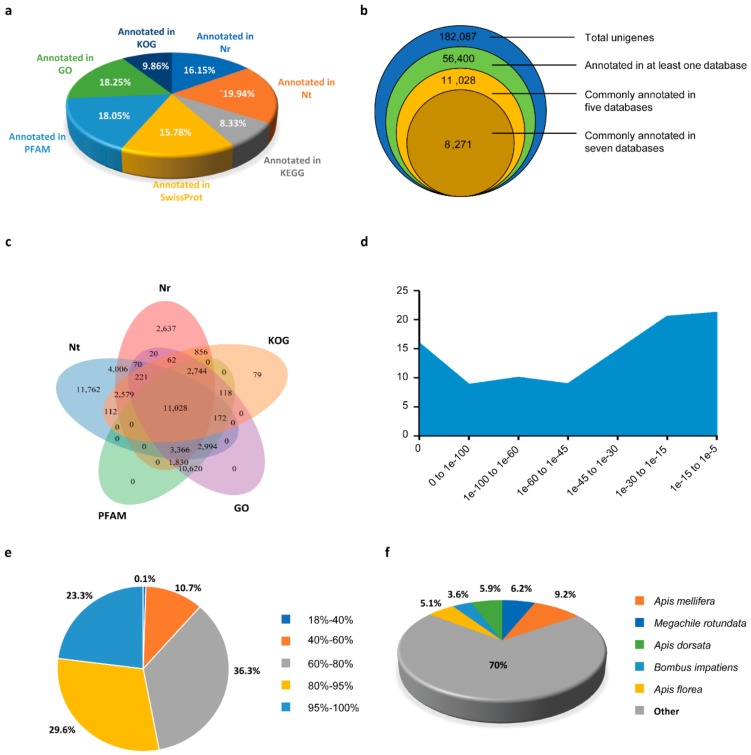
Functional annotation of unigenes from *Vespa velutina* transcriptome. (**a**) Summary of the annotation by the seven databases. Nr, NCBI non-redundant protein sequences; Nt, NCBI nucleotide sequences; PFAM, Protein family; KOG, euKaryotic Ortholog Groups; SwissProt, UniProt Knowledgebase; KEGG, Kyoto Encyclopedia of Genes and Genomes; GO, Gene Ontology; (**b**) Statistics of the number of unigenes annotated by single or multiple databases; (**c**) Venn diagram for annotations in the five databases; (**d**) E-value distribution of BLAST hits against the Nr database; (**e**) Similarity distributions of the top BLAST hits against the Nr database; (**f**) Species distribution of the top BLAST hits against the Nr database.

To determine comprehensive functional information of the transcripts obtained, we conducted sequence similarity search using BLAST against seven public databases: NCBI non-redundant protein sequences (Nr); NCBI nucleotide sequences (Nt); Protein family (PFAM); UniProtKB/Swiss-Prot (SwissProt); Gene Ontology (GO); euKaryotic Ortholog Groups (KOG); and the Kyoto Encyclopedia of Genes and Genomes (KEGG) databases. In total, 182,087 unigenes were successfully annotated to all the databases with the annotation ratios varying from 8.33% to 19.94% (Figure 2a,b). Significantly, 30.97% of the annotated unigenes were matched to at least one database, and 4.54% of the annotated unigenes were commonly annotated in all seven databases (Figure 2b). A Venn diagram showed that 6.06% of the annotated unigenes were commonly mapped to the five major databases, including Nr, Nt, PFAM, KOG, and GO (Figure 2c). Due to the limited data for the *Vespa velutina* genome, a large amount of unigenes were uncharacterized, but these results provide resources for further gene identification in *Vespa velutina*. The annotation data were shown in Additional files 3–9 (Tables S7–S13).

We performed similarity identification of unigenes against the Nr database with the threshold of E-value ≤ 1 × 10^−5^; the majority of the unigenes (~60%) were distributed ranging from 0 to 1 × 10^−30^. The most common E-value distribution ranged from 1 × 10^−15^ to 1 × 10^−5^ and accounted for 21.2% of unigenes (Figure 2d). Additionally, 99.9% of unigenes were matched to sequences in the NCBI with similarities greater than 40%, and 52.9% of unigenes were mapped with similarities greater than 80% (Figure 2e). The BLAST results showed that *Vespa velutina* unigenes had similarity to a wide range of species, and 9.2% of unigenes had maximum similarity with *Apis mellifera* (Figure 2f). 

### 3.3. Functional Classification in GO, KOG, and KEGG Databases

**Figure 3:**
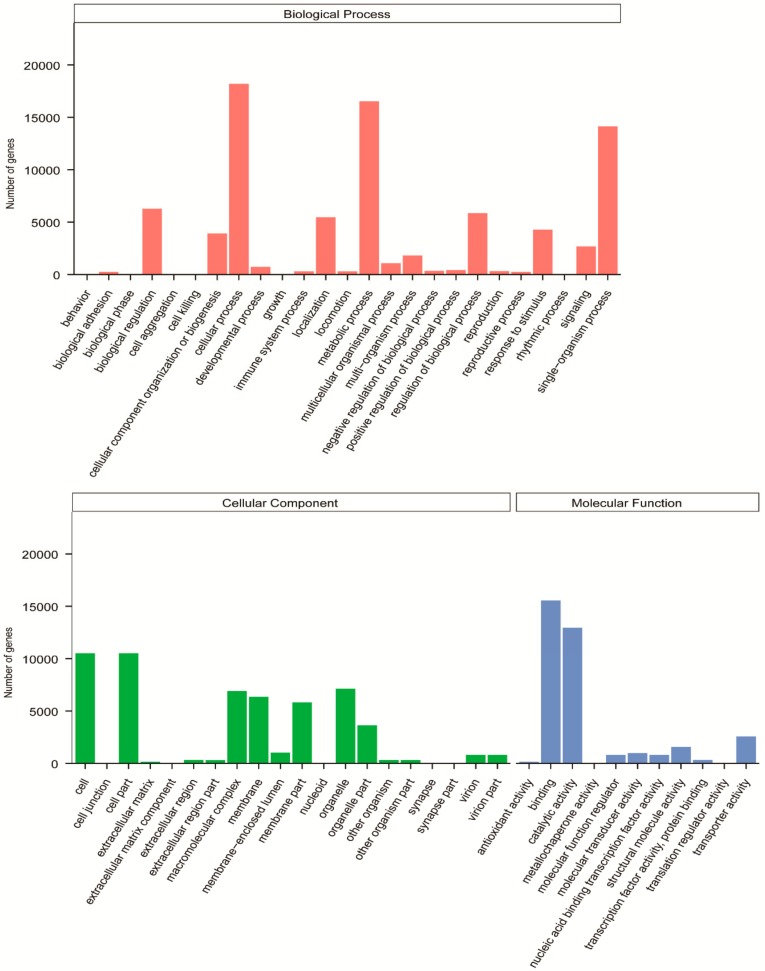
Functional annotation and classification of unigenes from *Vespa velutina* transcriptome in Gene Ontology (GO).

**Figure 4:**
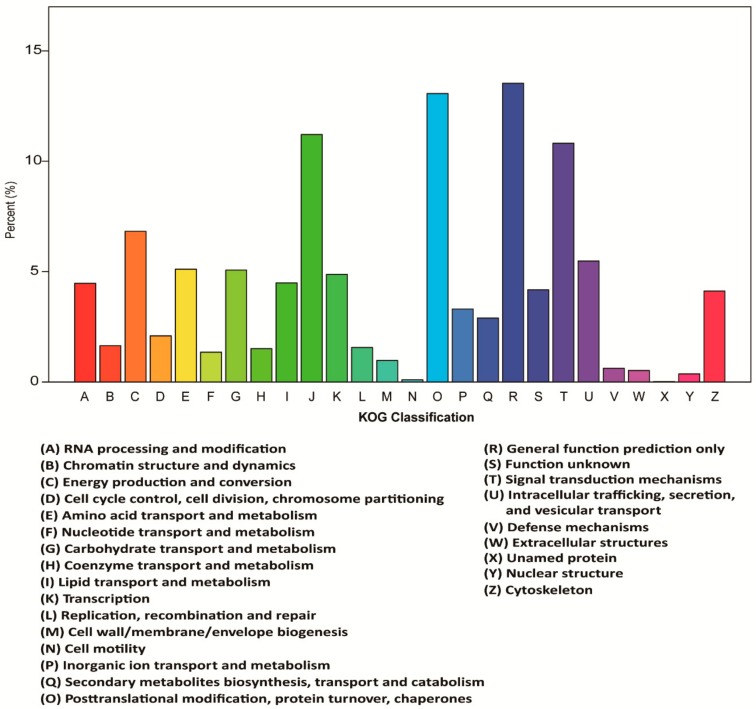
Functional annotation and classification of unigenes from *Vespa velutina* transcriptome in euKaryotic Ortholog Groups (KOG).

**Figure 5:**
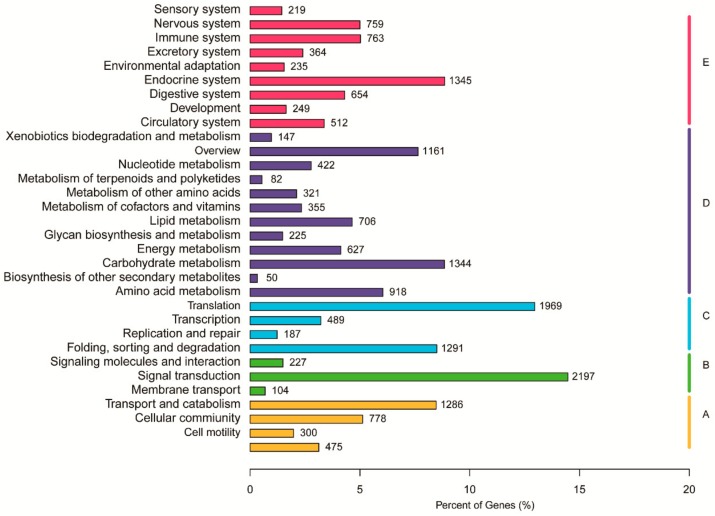
Functional annotation and classification of unigenes from *Vespa velutina* transcriptome in Kyoto Encyclopedia of Genes and Genomes (KEGG). Five categories were observed: A, cellular processes; B, environmental information processing; C, genetic information processing; D, metabolism; and E, organismal systems.

To further classify predicted functions of *Vespa velutina* genes, we investigated the detailed functional classification of all the unigenes in GO, KOG, and KEGG databases. Based on sequence homology, 33,245 unigenes were categorized into 56 functional groups in the GO database (Figure 3). The three main categories were biological process, cellular component, and molecular function, and they had the following occupancy: “cellular process” (18,198; 54.74% of 33,245); “cell part” (10,515; 31.63% of 33,245), and “binding” (15,543; 46.75% of 33,245) (Figure 3). In addition, a total of 17,971 unigenes were assigned appropriate KOG clusters (Figure 4). Among the 26 KOG categories, the cluster for “general function prediction” represented the largest group (2433; 13.54% of 17,971), and “cell motility” (66; 0.37% of 17,971) was the smallest group (Figure 4). In total, 15,184 unigenes were significantly matched to the KEGG database and assigned to 32 KEGG pathways (Figure 5). The major pathways were “signal transduction” (2197; 14.50% of 15,184), “translation” (1969; 12.97% of 15,184), and “endocrine system” (1345; 8.86% of 15,184) (Figure 5). The classification data was shown in Additional files 10–12 (Tables S14–S16).

### 3.4. Development of SSR and SNP Markers

**Figure 6:**
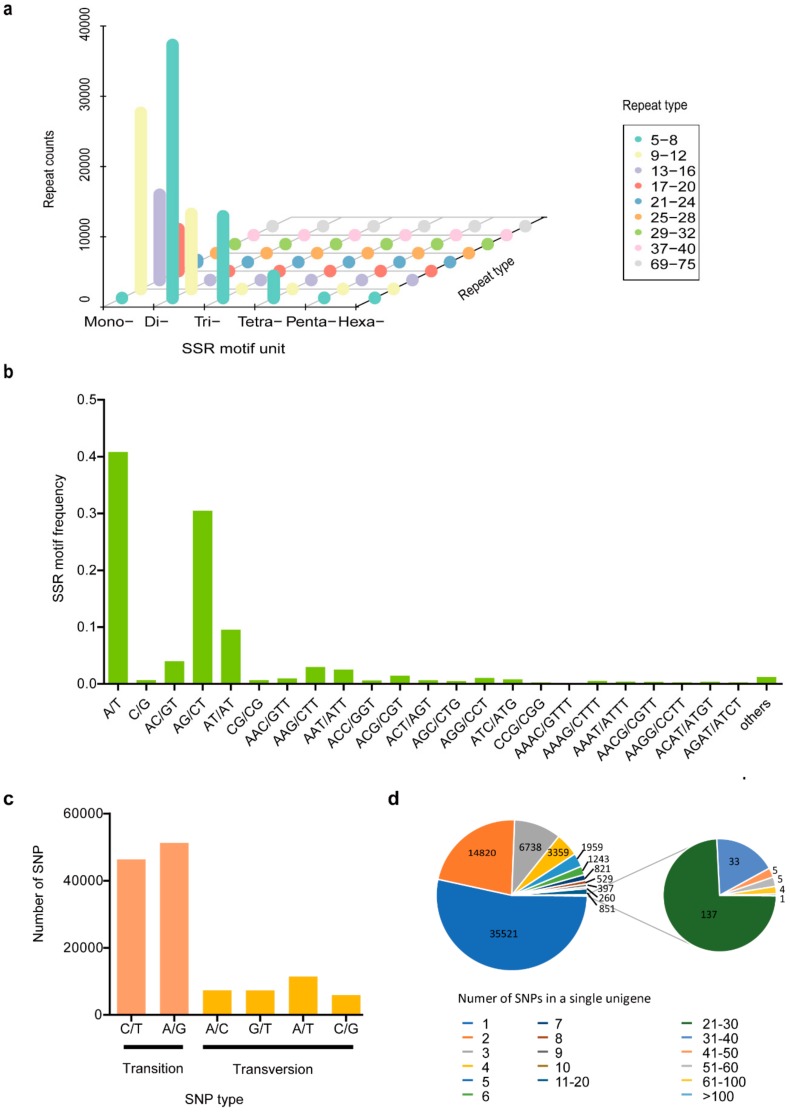
Characterization of the Simple Sequence Repeats (SSRs) and Single Nucleotide Polymorphisms (SNP) markers. (**a**) Distribution of the SSR motifs; (**b**) Frequencies of the classified SSR motifs; (**c**) Classification of SNPs and corresponding numbers; (**d**) The number of SNP-containing unigenes with different numbers of SNPs in a single unigene.

In total, 105,097 SSRs were obtained from 182,087 unigenes (88,549,722 bp) assembled from the *Vespa velutina* transcriptome (Table S17: Additional file 13). A total of 22,624 unigene sequences contained more than one SSR, and 5783 SSRs were present in compound form. As shown in Table S18 and Figure 6a, the dinucleotide repeat motif was the most abundant, accounting for 44.45% of the SSRs, followed by the mononucleotide (41.37%) and trinucleotide (11.07%) repeat motifs. We detected the frequencies of the classified SSR motifs, and found that the A/T motif was the most abundant (40.76%), followed by the AG/CT (30.43%) and the AT/TA motif (9.47%) (Figure 6b). In the A/T type, ten tandem iterations accounted for the highest frequency, while the AG/CT motif with eight tandem iterations was found to be the most common (Figure 6b). Subsequently, pairs of primers could be designed for 13,602 SSR loci using Primer 3 (v.2.3.5) software with default parameters, and three pairs of primers were designed for each SSR loci. The detail information of primers was provided in Additional file 14 (Table S19). 

In total, 129,387 SNPs were discovered, among them, about 30% within CDS and ~70% lie in untranslated regions (Tables S20 and S21). Of these SNPs, approximately 20% were synonymous mutants while approximately 7% were nonsynonymous mutants (Tables S20 and S21). The numbers of transition (Ts) and transversion (Tv) type SNPs were 97,471 (75%) and 31,916 (25%), respectively, with a Ts/Tv ratio of 3.05 (Figure 6c). Most of the SNPs were A/G (35.76%) type followed by T/C (39.57%) (Figure 6c). In the case of transversion substitution class, the frequency of occurrence of the SNPs was as follows: T/A (8.83%) and A/C (5.67%), followed by G/T (5.62%) and G/C (4.55%) (Figure 6c). In addition, among all the SNP-containing unigenes, about a half (53.3%) have single SNP marker, ~1.6% have more than 10 SNPs, and only one unigene contain more than 100 SNPs (Figure 6d, Table S22: Additional file 16). The highest number of SNPs (280) was found in the RNA-dependent RNA polymerase unigene (Table S22: Additional file 16). Among the top 30 number of SNPs contained in a single unigene, a total of 1655 SNPs were found in 30 transcripts which were involved in transcription regulation, DNA and RNA methylation, defense response, and oxidation-reduction process (Table S22: Additional file 16). Two unigenes, which were all belonged to Cytochrome P450 family, contained 59 and 40 SNPs, respectively (Table S22: Additional file 16).

### 3.5. Identification of Potential Target Genes of OBP and Vitellogenin in the Hornet Brain

**Figure 7:**
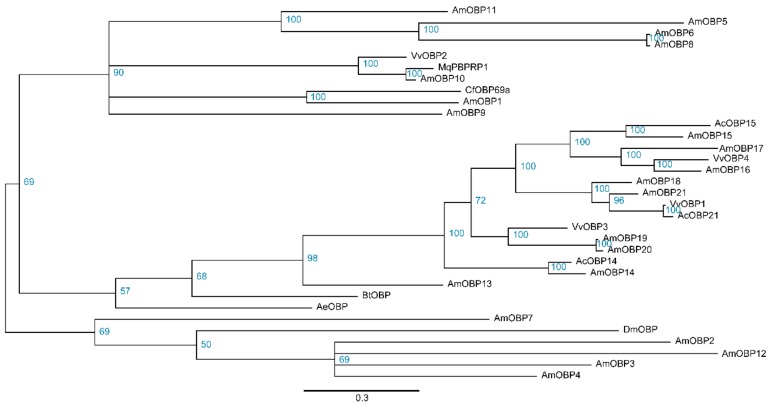
Phylogenetic analysis of insect odorant binding proteins (OBPs) in the brain of *Vespa velutina* and other seven insects. Vv, *Vespa velutina*; Am, *Apis mellifera*; Ac, *Apis cerana*; Mq, *Melipona quadrifasciata*; Bt, *Bombus terrestris*; Ae, *Acromyrmex echinatior*; Cf, *Camponotus floridanus*; Dm, *Drosophila melanogaster*.

**Figure 8:**
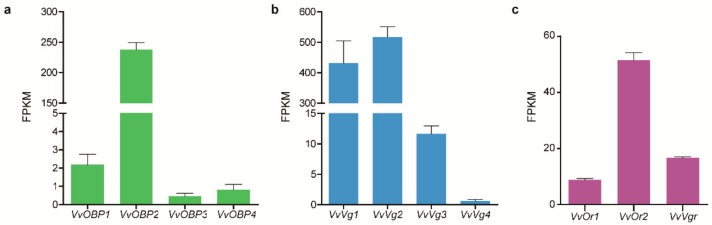
Gene expression detection of *VvOBP1-4*, *VvVg1-4*, *VvOr1-2*, and *VvVgr* in the brain of *Vespa velutina*. (**a**) *VvOBP1-4* expression level; (**b**) *VvVg1-4* expression level; (**c**) Gene expression level of *VvOr1-2* and *VvVgr*. The y axis indicates the mean ± SD of FPKM (fragments per kilobase million) from six samples.

**Figure 9:**
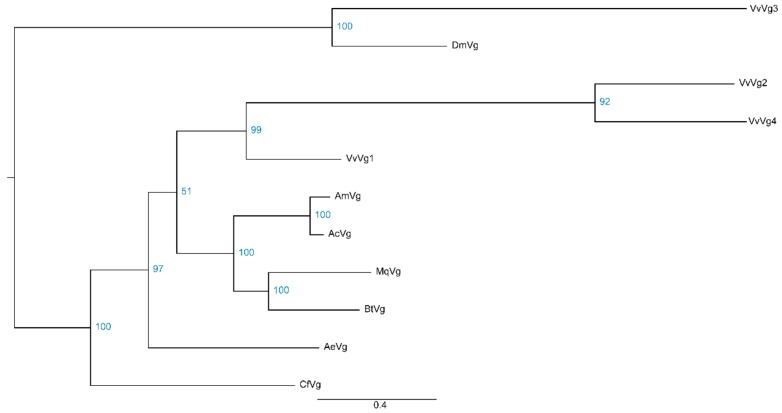
Phylogenetic analysis of of insect vitellogenins (Vgs) in the brain of *Vespa velutina* and other seven insects. Vv, *Vespa velutina*; Am, *Apis mellifera*; Ac, *Apis cerana*; Mq, *Melipona quadrifasciata*; Bt, *Bombus terrestris*; Ae, *Acromyrmex echinatior*; Cf, *Camponotus floridanus*; Dm, *Drosophila melanogaster*.

The disruption of insect behavior could suggest novel strategies for controlling this pest. Genes encoding OBP and vitellogenin have been shown to play essential roles in regulating insect behavior [28,29,30], and these genes were identified in our transcriptome data to be potential target genes. Firstly, the gene expression levels were estimated by RSEM based on *de novo* transcriptome assemblies, then putative homologs were developed from these expressed genes. In total, four *OBP* and two *OBP receptor* genes were identified and named as *VvOBP1-4* and *VvOr1-2*, respectively. The bayesian phylogenetic trees of the VvOBPs and VvOrs were constructed using six Hymenoptera species and *Drosophila melanogaster*. It is demonstrated that VvOBPs were mainly clustered with OBPs from *Apis mellifera* and *Apis cerana* (Figure 7), and VvOr1-2 were clustered with Ors from *Apis cerana*, *Bombus terrestris*, *Acromyrmex echinatior*, and *Camponotus floridanus* (Figure S1). The expression profiles of all *VvOBP* and *VvOr* genes measured by FPKM (fragments per kilobase million) values showed that *VvOBP2* was expressed at an extremely high level in the hornet brain, and the expression level of *VvOr2* was higher than *VvOr1* and other *VvOBPs* (Figure 8). In total, four *Vgs* (*VvVg1-4*) and one *Vg receptor* (*VvVgr*) were identified from the hornet transcriptome. In the phylogenetic analysis, VvVg2-4 were clustered together and VvVg1 was clustered with *Drosophila melanogaster* Vg (Figure 9). And VvVgr was clustered with Vgr from bees, including *Apis mellifera*, *Apis cerana*, *Melipona quadrifasciata*, and *Bombus terrestris* (Figure S2). VvVg1 and VvVg2 were highly expressed in the hornet brain, while VvVg3, VvVg4, and VvVgr were expressed much lower (Figure 8).

## 4. Discussion

In recent years, *Vespa velutina* invasions have drawn sufficient attention to warrant their control due to their worldwide negative impacts. Although methods are under development to solve the hornet invasion problem, effective means for controlling this species have rarely been achieved. The transcriptome data of the hornet brain in our results provides potential strategies for hornet control at the molecular level.

It is accepted that a targeted pest management strategy is more effective than non-specific control. The availability of targeted treatment primarily relies on the proper choice of a target gene [39], thus our study aimed to provide transcriptome data and target gene resources. The annotation of transcripts in our results illustrate various functional genes involved in multiple pathways, including development, reproductive, sensory, nervous, digestive and immune system. Moreover, the genes encoding OBPs and vitellogenin were selected from expression profiles, and these homologs were reported to be essential in foraging, predation, social organization, and reproductive behavior [28,29,30], which are potential candidates of targets in biological hornet control. Finally, the SNP and SSR markers can be used for identification and mapping of functional genes that would be selected as target loci.

Molecular markers have contributed significantly to the understanding of genetic diversity, ecology, and mapping of important genes [40]. SSR and SNP markers are gaining popularity among researchers because of their high reproducibility, co-dominant nature, high polymorphic nature, and their high number of alleles per locus [40]. However, the development of these markers is costly and difficult in a species lacking genome information, such as *Vespa velutina*. In *Vespa velutina*, mitochondrial DNA sequencing [20,21,22] and microsatellite-enriched libraries [23] have been constructed and have aided the development of mtDNA and microsatellite markers used in the detection of hornet invasion, but these molecular markers are small scale and not very suitable for evolutionary and phylogenetic studies [41]. Our transcriptome data has provided various and reliable marker resources on the genome-wide scale. The large SSR and SNP data sets in our study can be used in *Vespa velutina* hornet control; these data can be used for DNA phenotyping identification [42], introgression estimation [43], and dispersal analyses of invasive pest [44]. They also can be used extend the study of *Vespa velutina* to evolution [45], molecular ecology [46], polymorphism, and genetic diversity [47]. 

## 5. Conclusions

The present study represents the first transcriptome analysis for the brain of *Vespa velutina*. These data has been shown to be of high quality and provides valuable resources: (1) more than 41 million clean reads were generated in each sample and were assembled to 182,087 meaningful unigenes; (2) 56,400 unigenes were annotated to seven databases and assigned to a multitude of important physiological pathways; (3) a set of reliable SSR and SNP markers were obtained for investigation of *Vespa velutina*; (4) the homologous genes encoding crucial behavior regulation factors, OBPs and vitellogenin, were identified from highly expressed transcripts. These data collective provide abundant functional gene targets and markers for the invasion control and molecular study of *Vespa velutina*.

## Supplementary Materials

The following are available online at https://www.mdpi.com/2075-4450/11/2/101/s1, Figure S1. Phylogenetic analysis of insect odorant receptors (Ors) and other seven insects, Figure S2. Phylogenetic analysis of insect vitellogenin receptor (Vgr) and other six insects, Table S1. Database, software, and parameters used in annotation for the *Vespa velutina* transcriptome, Table S2. Summary of the sequenced data for the *Vespa velutina* transcriptome, Table S3. The assembled Trinity dataset, Table S4. The assembled unigene dataset, Table S5. Summary of the *de novo* assembled data for the *Vespa velutina* transcriptome, Table S6. The results of BUSCO analysis, Table S7. The annotation of the *Vespa velutina* transcriptome in Nr database, Table S8. The annotation of the *Vespa velutina* transcriptome in Nt database, Table S9. The annotation of the *Vespa velutina* transcriptome in PFAM database, Table S10. The annotation of the *Vespa velutina* transcriptome in SwissProt database, Table S11. The annotation of the *Vespa velutina* transcriptome in Gene Ontology (GO) database, Table S12. The annotation of the *Vespa velutina* transcriptome in euKaryotic Ortholog Groups (KOG) database, Table S13. The annotation of the *Vespa velutina* transcriptome in KEGG ORTHOLOG (KO) database, Table S14. Functional classification of unigenes from *Vespa velutina* transcriptome in GO, Table S15. Functional classification of unigenes from *Vespa velutina* transcriptome in KOG, Table S16. Functional classification of unigenes from *Vespa velutina* transcriptome in Kyoto Encyclopedia of Genes and Genomes (KEGG), Table S17. Simple sequence repeats (SSRs) motifs and corresponding frequencies, Table S18. Distribution of SSRs among the *Vespa velutina* unigenes based on the number of repeat units, Table S19. SSR primers, Table S20. Single Nucleotide Polymorphisms (SNP) detected in the *Vespa velutina* transcriptome, Table S21. SNP detection among the *Vespa velutina* unigenes, Table S22. SNP annotation.
