# Supplementary material for: De Novo Transcriptomic Resources in the Brain of Vespa velutina for Invasion Control
Source: Insects. 2020 Feb 3;11(2):101. doi: 10.3390/insects11020101 (PMC7074412; doi:10.3390/insects11020101)
Supplement: Supplementary file 1 [file insects-11-00101-s001.zip › Supplement information/insects-672026-supplement information-revision.docx]

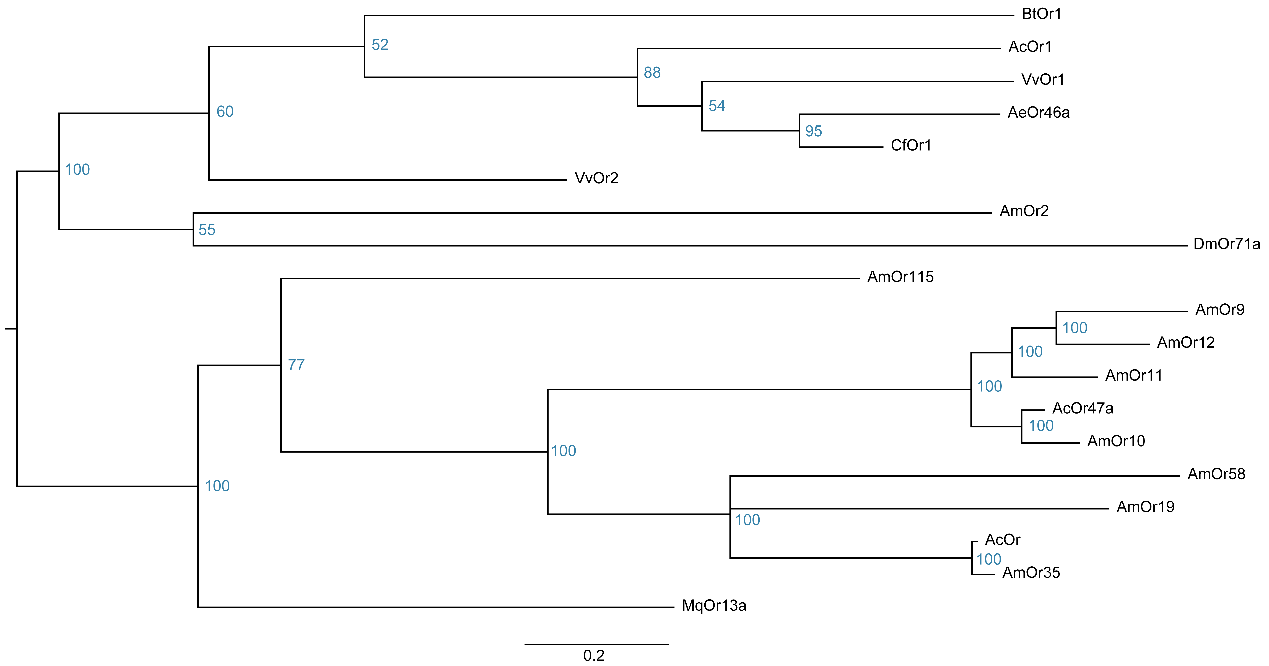


**Figure S1.** Phylogenetic analysis of insect odorant receptors (Ors) and other seven insects. Vv, *Vespa velutina*; Am, *Apis mellifera*; Ac, *Apis cerana*; Mq, *Melipona quadrifasciata*; Bt, *Bombus terrestris*; Ae, *Acromyrmex echinatior*; Cf, *Camponotus floridanus*; Dm, *Drosophila melanogaster*.


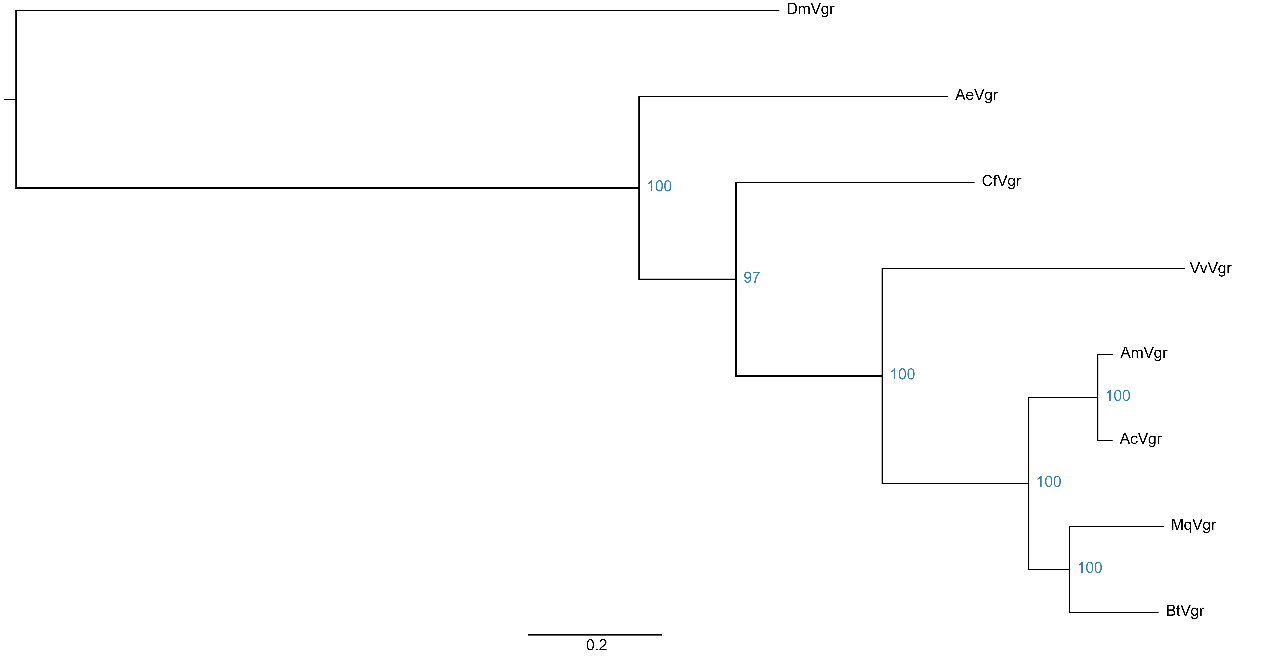


**Figure S2.** Phylogenetic analysis of insect vitellogenin receptor (Vgr) and other six insects. Vv, *Vespa velutina*; Am, *Apis mellifera*; Ac, *Apis cerana*; Mq, *Melipona quadrifasciata*; Bt, *Bombus terrestris*; Ae, *Acromyrmex echinatior*; Cf, *Camponotus floridanus*; Dm, *Drosophila melanogaster*.

**Table S1.** Database, software, and parameters used in annotation for the *Vespa velutina* transcriptome

| **Data bases** | **Name** | **Software** | **Parameters** |
| --- | --- | --- | --- |
| Nr | NCBI non-redundant protein sequences | NCBI blast 2.2.28+ | E-value = 1e-5 |
| Nt | NCBI nucleotide sequences | NCBI blast 2.2.28+ | E-value = 1e-5 |
| PFAM | Protein family | HMMER 3.0 package,  hmmscan | E-value = 0.01 |
| SwissProt | UniProt Knowledgebase | NCBI blast 2.2.28+ | E-value = 1e-5 |
| GO | Gene Ontology | Blast2GO v2.5 | E-value = 1e-6 |
| KOG | euKaryotic Ortholog Groups | NCBI blast 2.2.28+ | E-value = 1e-3 |
| KEGG | Kyoto Encyclopedia of Genes and Genomes | KEGG Automatic  Annotation Server | E-value= 1e-10 |

**Table S2.** Summary of the sequenced data for the *Vespa velutina* transcriptome.

| Terms | V1 | V2 | V3 | V4 | V5 | V6 |
| --- | --- | --- | --- | --- | --- | --- |
| Raw reads | 53,110,162 | 59,156,920 | 54,111,586 | 47,222,548 | 47,569,698 | 61,311,504 |
| Raw data (bp) | 7,966,524,300 | 8,873,538,000 | 8,116,737,900 | 7,083,382,200 | 7,135,454,700 | 9,196,725,600 |
| Clean Reads | 42,675,086 | 46,981,420 | 43,274,368 | 41,158,562 | 41,329,702 | 53,453,034 |
| Clean data (bp) | 6,401,262,900 | 7,047,213,000 | 6,491,155,200 | 6,173,784,300 | 6,199,455,300 | 8,017,955,100 |
| Q30 | 91.51 | 90.98 | 91.53 | 94.69 | 94.64 | 94.63 |
| GC(%) | 37.36 | 37.34 | 38.03 | 37.99 | 37.72 | 37.37 |

**Table S3.** The assembled Trinity dataset.

See Additional file 1

**Table S4.** The assembled Unigene dataset.

See Additional file 2

**Table S5.** Summary of the *de novo* assembled data for the *Vespa velutina* transcriptome.

|  | Min Length | Mean Length | Median Length | Max Length | N50 | N90 | Total Nucleotides |
| --- | --- | --- | --- | --- | --- | --- | --- |
| Transcripts | 201 | 675 | 320 | 26255 | 1315 | 256 | 140684525 |
| Unigenes | 201 | 486 | 302 | 26255 | 522 | 236 | 88549722 |

**Table S6.** The results of BUSCO analysis.

| Type | Complete (single) | | Complete (duplicated) | | | Fragmented | | | Missing | | | |
| --- | --- | --- | --- | --- | --- | --- | --- | --- | --- | --- | --- | --- |
|  | Number | Percent | Number | Percent | | Number | Percent | | Number | | Percent | |
| Trinity. fasta | 491 | 50.2 % | 480 | | 49.1 % | 4 | | 0.4 % | 3 | | | 0.3 % |
| unigene. fasta | 894 | 91.4 % | 58 | | 5.9 % | 18 | | 1.8 % | 8 | 0.9 % | | |

**Table S7.** The annotation of the *Vespa velutina* transcriptome in Nr database.

See Additional file 3

**Table S8.** The annotation of the *Vespa velutina* transcriptome in Nt database.

See Additional file 4

**Table S9.** The annotation of the *Vespa velutina* transcriptome in PFAM database.

See Additional file 5

**Table S10.** The annotation of the *Vespa velutina* transcriptome in SwissProt database.

See Additional file 6

**Table S11.** The annotation of the *Vespa velutina* transcriptome in Gene Ontology (GO) database.

See Additional file 7

**Table S12.** The annotation of the *Vespa velutina* transcriptome in euKaryotic Ortholog Groups (KOG) database.

See Additional file 8

**Table S13.** The annotation of the *Vespa velutina* transcriptome in KEGG ORTHOLOG (KO) database.

See Additional file 9.

**Table S14.** Functional classification of unigenes from Vespa velutina transcriptome in GO.

See Additional file 10

**Table S15.** Functional classification of unigenes from Vespa velutina transcriptome in KOG.

See Additional file 11

**Table S16.** Functional classification of unigenes from Vespa velutina transcriptome in Kyoto Encyclopedia of Genes and Genomes (KEGG).

See Additional file 12

**Table S17.** Simple sequence repeats (SSRs) motifs and corresponding frequencies.

See Additional file 13

**Table S18.** Distribution of SSRs among the *Vespa velutina* unigenes based on the number of repeat units.

|  | **5** | **6** | **7** | **8** | **9** | **10** | **11** | **≥12** | **Total** | **%** |
| --- | --- | --- | --- | --- | --- | --- | --- | --- | --- | --- |
| Mononucleotide | 0 | 0 | 0 | 0 | 0 | 12,877 | 7,026 | 23,574 | 43,477 | 41.37 |
| Dinucleotide | 0 | 12,083 | 11,696 | 12,235 | 8,349 | 2,076 | 263 | 11 | 46,713 | 44.45 |
| Trinucleotide | 5,212 | 4,510 | 1,882 | 29 | 2 | 0 | 0 | 0 | 11,635 | 11.07 |
| Tetranucleotide | 3,079 | 88 | 2 | 3 | 2 | 1 | 0 | 2 | 3,177 | 3.02 |
| Pentanucleotide | 21 | 4 | 5 | 7 | 5 | 1 | 0 | 1 | 44 | 0.04 |
| Hexanucleotide | 11 | 8 | 4 | 5 | 3 | 3 | 2 | 0 | 36 | 0.03 |

**Table S19.** SSR primers.

See Additional file 14

**Table S20.** Single Nucleotide Polymorphisms (SNP) detected in the *Vespa velutina* transcriptome.

See Additional file 15

**Table S21.** SNP detection among the *Vespa velutina* unigenes

| **Sample** | **Totle**  **SNP** | **Noncoding SNP** | | **Coding SNP** | | **Synonymous** | | **Nonsynonymous** | |
| --- | --- | --- | --- | --- | --- | --- | --- | --- | --- |
|  |  | **Number** | **Percent** | **Number** | **Percent** | **Number** | **Percent** | **Number** | **Percent** |
| V1 | 54,476 | 17,487 | 67.90% | 17,487 | 32.10% | 13,245 | 24.31% | 4,242 | 7.79% |
| V2 | 52,477 | 15,699 | 70.08% | 15,699 | 29.92% | 11,632 | 22.17% | 4,067 | 7.75% |
| V3 | 51,854 | 16,693 | 67.81% | 16,693 | 32.19% | 12,455 | 24.02% | 4,238 | 8.17% |
| V4 | 48,017 | 13,859 | 71.14% | 13,859 | 28.86% | 10,722 | 22.33% | 3,137 | 6.53% |
| V5 | 47,872 | 12,515 | 73.86% | 12,515 | 26.14% | 9,518 | 19.88% | 2,997 | 6.26% |
| V6 | 49,862 | 11,336 | 77.27% | 11,336 | 22.73% | 8,646 | 17.34% | 2,690 | 5.39% |

**Table S22.** SNP annotation

See Additional file 16
