# Supplementary figures and images for: De Novo Transcriptomic Resources in the Brain of Vespa velutina for Invasion Control
Source: Insects. 2020 Feb 3;11(2):101. doi: 10.3390/insects11020101 (PMC7074412; doi:10.3390/insects11020101)

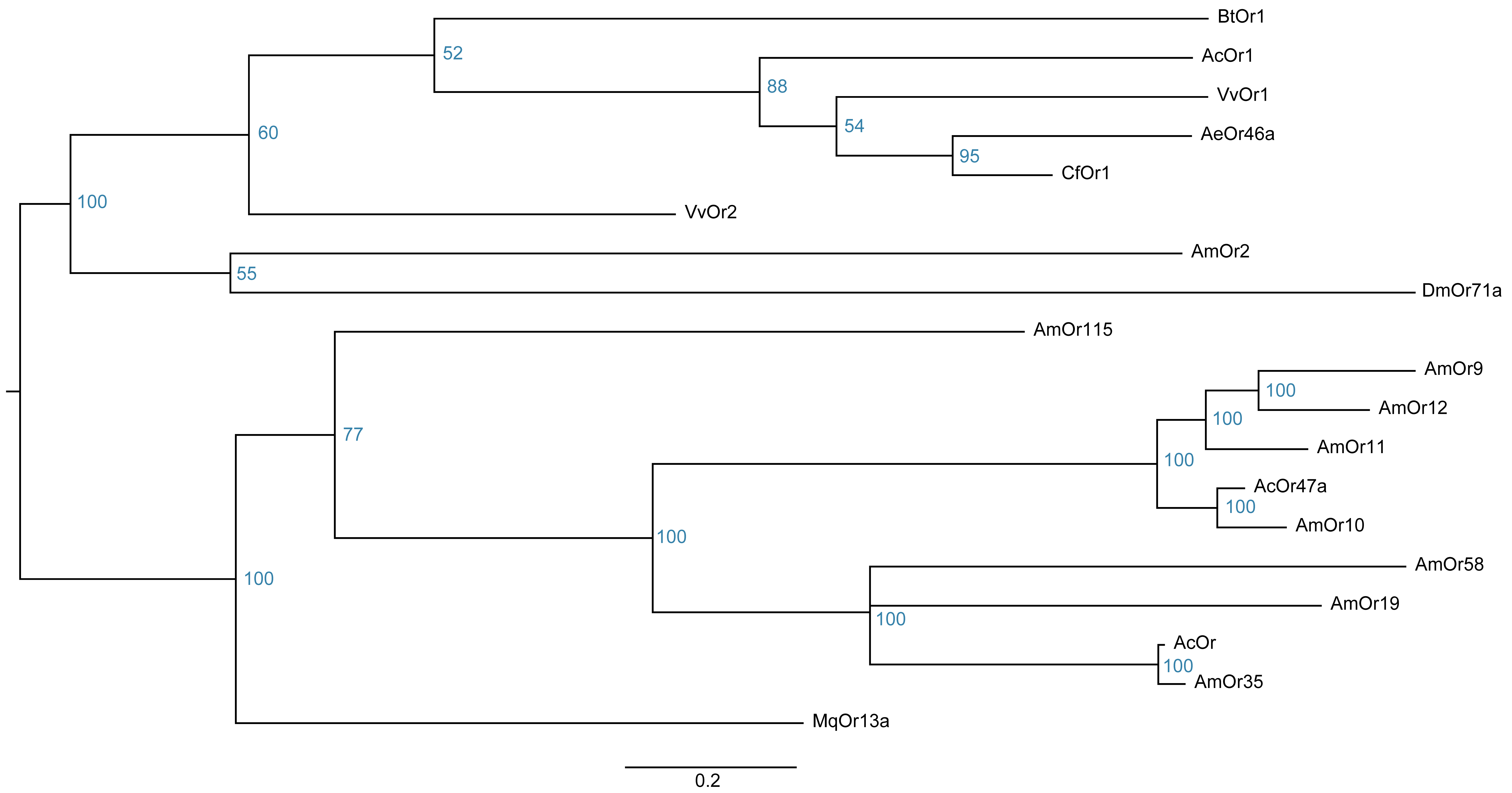

Supplement: Supplementary file 1 [file insects-11-00101-s001.zip › Supplement information/Figure S1.tif]

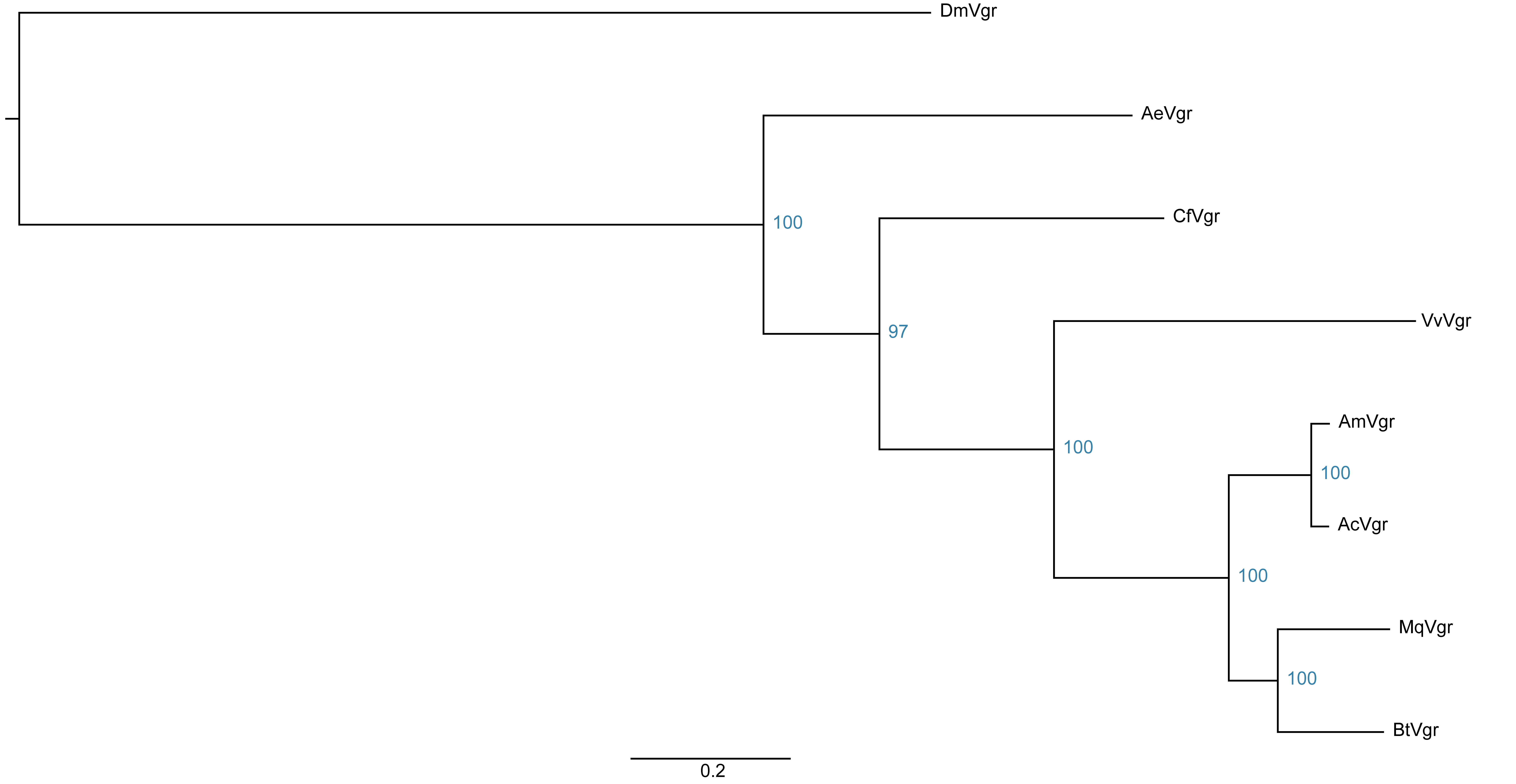

Supplement: Supplementary file 1 [file insects-11-00101-s001.zip › Supplement information/Figure S2.tif]
